# No association between thickening fraction of the diaphragm and extubation success in ventilated children

**DOI:** 10.3389/fped.2023.1147309

**Published:** 2023-03-24

**Authors:** Anita Duyndam, Joke Smit, Robert Jan Houmes, Leo Heunks, Jeroen Molinger, Marloes IJland, Joost van Rosmalen, Monique van Dijk, Dick Tibboel, Erwin Ista

**Affiliations:** ^1^ Pediatric Intensive Care, Department of Pediatric Surgery, Erasmus MC-Sophia Children's Hospital, University Medical Center Rotterdam, Rotterdam, Netherlands; ^2^ Intensive Care Adults, Erasmus MC, University Medical Center Rotterdam, Rotterdam, Netherlands; ^3^ Division of Critical Care, Department of Anesthesiology, Duke University School of Medicine, Durham, NC, United States; ^4^ Department of Intensive Care Medicine, Radboud University Medical Center, Radboud Institute for Health Sciences, Nijmegen, Netherlands; ^5^ Department of Biostatistics, Erasmus MC, University Medical Center Rotterdam, Rotterdam, Netherlands; ^6^ Department of Epidemiology, Erasmus MC, University Medical Center Rotterdam, Rotterdam, Netherlands

**Keywords:** mechanical ventilation, ultrasound, diaphragm, thickening fraction, pediatric

## Abstract

**Introduction:**

In mechanically ventilated adults, thickening fraction of diaphragm (dTF) measured by ultrasound is used to predict extubation success. Whether dTF can also predict extubation success in children is unclear.

**Aim:**

To investigate the association between dTF and extubation success in children. Second, to assess diaphragm thickness during ventilation and the correlation between dTF, diaphragm thickness (Tdi), age and body surface.

**Method:**

Prospective observational cohort study in children aged 0–18 years old with expected invasive ventilation for >48 h. Ultrasound was performed on day 1 after intubation (baseline), day 4, day 7, day 10, at pre-extubation, and within 24 h after extubation. Primary outcome was the association between dTF pre-extubation and extubation success. Secondary outcome measures were Tdi end-inspiratory and Tdi end-expiratory and atrophy defined as <10% decrease of Tdi end-expiratory versus baseline at pre-extubation. Correlations were calculated with Spearman correlation coefficients. Inter-rater reliability was calculated with intraclass correlation (ICC).

**Results:**

Fifty-three patients, with median age 3.0 months (IQR 0.1–66.0) and median duration of invasive ventilation of 114.0 h (IQR 55.5–193.5), were enrolled. Median dTF before extubation with Pressure Support 10 above 5 cmH_2_O was 15.2% (IQR 9.7–19.3). Extubation failure occurred in six children, three of whom were re-intubated and three then received non-invasive ventilation. There was no significant association between dTF and extubation success; OR 0.33 (95% CI; 0.06–1.86). Diaphragmatic atrophy was observed in 17/53 cases, in three of extubation failure occurred. Children in the extubation failure group were younger: 2.0 months (IQR 0.81–183.0) vs. 3.0 months (IQR 0.10–48.0); *p* = 0.045. At baseline, pre-extubation and post-extubation there was no significant correlation between age and BSA on the one hand and dTF, Tdi- insp and Tdi-exp on the other hand. The ICC representing the level of inter-rater reliability between the two examiners performing the ultrasounds was 0.994 (95% CI 0.970–0.999). The ICC of the inter-rater reliability between the raters in 36 paired assessments was 0.983 (95% CI 0.974–0.990).

**Conclusion:**

There was no significant association between thickening fraction of the diaphragm and extubation success in ventilated children.

## Introduction

Many invasively ventilated children develop respiratory muscle weakness at the time of extubation, which is associated with prolonged PICU length of stay, prolonged invasive ventilation (MV) duration, and greater risk of death (1–3). Prolonged invasive MV carries the risk of ventilator-associated pneumonia and ventilator-induced lung injury; nevertheless, premature extubation should be prevented (4–7). The optimal timing of extubation readiness in children is still being debated (8, 9).

Diaphragmatic thickness (Tdi) and diaphragm thickening fraction (dTF) as assessed with ultrasound are widely used parameters to evaluate diaphragm function in mechanically ventilated patients with suspected diaphragm weakness. dTF has been correlated with diaphragm strength, and diaphragm thickening was not observed in patients with diaphragm paralysis (10, 11). Several adult studies have provided evidence of diaphragm dysfunction and frequent reintubation if the diaphragm cannot sufficiently thicken with a 19%–36% value of dTF cut-off (12–17). However, a new study of Poulard et al. found a weak relation between diaphragmatic strength and dTF (18).

Studies in ventilated children reported evidence of either a decrease or increase in Tdi after a period of invasive ventilation (19–23). In addition, Montoro et al. found a strong association between dTF and neuromuscular blockade (NMB). Glau et al. found a longer median length of MV in subjects exposed to NMB and a trend toward increased daily rates of atrophy. In this study, dTF was significantly correlated with spontaneous breathing *via* a support ventilation mode (19, 20). However, information on what this means in practice for failure of extubation is scarce (20, 24, 25). Lee et al. found a significantly different dTF post-extubation between the 28 children who were successfully extubated and the three children in whom extubation failed (*p* < 0.001) (24). Montoro et al. found no significant differences in dTF at baseline, pre-extubation and post-extubation between the 13 children with extubation failure and the 32 children with successful extubation (20). Abdel Rahman et al. found a cut-off value for dTF of ≥23.2% with an area under curve of 0.876 for predicting weaning success in 103 children, mostly infants (25). Xue et al. found that a cut-off value for dTF of ≥21% was associated with successful weaning, with a sensitivity of 0.82 and a specificity of 0.81 in 50 ventilated children with a median age of 36 months. The authors did not provide information about extubation failure (26).

We hypothesized that children with a low dTF would be prone to extubation failure and aimed to investigate the association between dTF before extubation and extubation success in ventilated children aged 0–18 years old. Furthermore, the presence of diaphragm atrophy and the correlation between dTF, end-inspiratory and end-expiratory diaphragm thickness (Tdi insp, Tdi exp) and age and body surface area (BSA) were investigated.

## Method

We performed an observational prospective cohort study from 1 September 2019 until 1 March 2021.

### Study population

We included children, aged 0–18 years old and invasively ventilated for more than 48 h admitted to a 28-bed ICU of a tertiary referral academic children's hospital with surgical, cardiac and medical beds in Rotterdam, the Netherlands. Excluded were patients after cardiac surgery because these patients are often ventilated for a short time; those who are longer ventilated often have pleural fluid or pericardial fluid affecting the diaphragm (27).

Informed consent from the parents of each participant was obtained.

### Ultrasound measurement of the diaphragm

Ultrasound of the diaphragm was performed with a Sonosite SII, portable system (Secma Holland) and with a Lumify portable ultrasound (Philips Nederland), with the child in a 30-degree supine position. We used the B mode (2-dimensional image) of the ultrasound. For details about the ultrasound measurement, see Additional File S1 and Supplementary Figure S1.

### Outcome measures

Primary outcome was the association between thickening fraction (measured on a support ventilation mode), expressed as the index of diaphragm thickness (dTF) before extubation, and extubation success. Extubation success was defined as no reintubation or non-invasive respiratory support required within 48 h of extubation. Only non-invasive ventilation counted as failure; high flow nasal cannula was not considered failure.

Secondary outcomes were diaphragm thickness at end-inspiration (Tdi insp) and at end-expiration (Tdi exp) during the period of ventilation (Additional File S1 Ultrasound measurement and Supplementary Figure S1), the presence of atrophy at the moment of extubation, the association between atrophy and extubation success, and intra-rater and inter-rater reliability. Atrophy was defined as a more than 10% decrease in end-expiratory Tdi at pre-extubation compared to end-expiratory Tdi at baseline (the first day of ventilation).

Other secondary outcomes were presence of respiratory support after extubation, ventilation condition [Pressure Control (PC), Pressure Regulated Volume Control (PRVC), Pressure Support (PS) Continuous Positive Airway Pressure (CPAP), PEEP, Pressure above PEEP], frequency rate and FiO_2_ at baseline on the first day of ventilation and just before extubation, respiratory support after extubation [non-invasive ventilation (NIV), high flow or low flow oxygen nasal cannula], corticosteroids before extubation or not, and dosages of sedatives and opiates (mcg/kg/hour) at time of extubation.

The following patient characteristics were collected: age, sex, weight, length, BSA, reason for admission, severity of illness (Pediatric Risk or Mortality Score III PRISM score), duration of ventilation, length of stay in the ICU (LOS) and reason for invasive ventilation.

### Study procedure

Ultrasounds were performed on fixed days: day 1 after intubation (between 24 and 48 h after intubation), day 4, day 7 and day 10 (depending on the duration of ventilation). In addition, ultrasounds were performed prior to extubation when the patient was already on a support mode or at the start of a SBT in a PS mode (PS 10 cmH_2_O above PEEP 5 cmH_2_O) and when applicable, just before extubation on CPAP with PEEP 5 (if SBT was successful). Finally, we made an ultrasound in the first 8 h after extubation with a maximum of 24 h after extubation as an outlier. For more details of the study procedure, see Additional File S2 with Supplementary Table S1.

The ventilation protocol, extubation criteria and sedation practice protocols are described in Additional File S3.

### Sample size calculation

Based on available literature in 2019, which concerned about 50 inclusions, we aimed at studying a convenience sample of fifty children (19, 20, 22).

### Statistical analysis

Descriptive continuous data are presented as mean (standard deviation) for normally distributed data, and median (IQR) for non-parametric data. Categorical variables are expressed as numbers and percentages. Thickening fraction (dTF) was compared between pre-extubation on pressure support, pre-extubation on CPAP (whenever possible) and post-extubation. Tdi was compared between baseline, pre-extubation on pressure support, pre-extubation on CPAP (whenever possible), and post-extubation.

These outcomes were also compared between patients who were successfully extubated and those who were not, on day one of ventilation, before extubation and after extubation. The association between dTF (independent variable) and extubation success (dependent variable) was analyzed with univariable logistic regression analysis. Normally distributed variables such as the differences between dTF and Tdi over time, patient characteristics, and dTF and Tdi between the extubation success group and extubation failure group were compared with the paired samples *t*-test and with the Mann-Whitney *U* test if these variables were not normally distributed. Categorical variables such as sex and reasons for admission were compared using chi-squared or Fisher exact tests.

We calculated the inter-rater reliability in terms of (1) performing the ultrasounds and (2) analyzing the recorded images. The procedure for calculating the inter-rater reliability in terms of performing the ultrasounds was as follows: each examiner independently made ultrasounds of 5 ventilated children 5 min after each other on the same day. This type of inter-rater reliability describes the variability between the two examiners when analyzing the thickness of the diaphragm of the recorded images of these 5 subjects performed simultaneously. AD analyzed these recorded images. The second type of inter-rater reliability, describes the level of agreement between independent raters who assessed the images. This metric was calculated from measurements of Tdi at end-inspiration and at end-expiration of 36 randomly selected ultrasound images. These images were assessed at two different times by two raters independently (AD and JS), without knowing each other’s results. Inter-rater reliability of the results was assessed using the intraclass correlation coefficient (ICC). The calculation of the ICC was based on a two-way mixed model with a consistency definition, reporting single measures. An ICC value below 0.5 is considered to indicate poor reliability, between 0.5 and 0.75 moderate reliability, between 0.75 and 0.9 good reliability, and any value above 0.9 excellent reliability (28).

A Spearman correlation coefficient (*r*_s_) with 95% confidence interval was calculated between body surface area (BSA) and diaphragm thickness at baseline, before and after extubation. The *r*_s_ between age in months and diaphragm thickness was calculated as well. For dTF the same procedure was applied without inclusion of the baseline values because there is no or little difference in thickness between inspiration and expiration in children who do not spontaneously breathe on the first day of ventilation. Missing data were pairwise deleted. A *p*-value of <0.05 was considered statistically significant. Data was analyzed with IBM SPSS version 25.0.

### Ethical considerations

The Medical Ethics Review Board of the Erasmus MC Rotterdam approved the study (MEC-2019-0365) and found the study not required to comply with the Medical Research Act, owing to its noninvasive nature. The study was performed in accordance with the ethical standards of the Declaration of Helsinki.

## Results

Fifty-three patients were enrolled, in whom a total of 226 ultrasounds were acquired, with an average of 4 per patient. Numbers of inclusion, exclusion and numbers of ultrasounds per patient are shown in Supplementary Figure S2. Demographic characteristics of these patients are shown in Supplementary Table S2. For various reasons some scheduled ultrasounds were not performed. We missed six ultrasounds, one for a patient at baseline, three at pre-extubation and two post-extubation. With regard to the latter, in the absence of the investigator (AD or JS), one extubation was performed before the ultrasound could be made and one was re-intubated before the ultrasound was made.

The ICC representing the level of inter-rater reliability between the two examiners performing the ultrasounds was 0.994 (95% CI 0.970–0.999). The ICC of the inter-rater reliability between the raters in 36 paired assessments was 0.983 (95% CI 0.974–0.990).

## Primary outcome

Analysis did not reveal a significant association between dTF and extubation success; OR was 0.33 (95% CI; 0.06–1.86). At both pre-extubation and post-extubation, dTF was not significantly different between the failure group and the success group (Supplementary Table S3).

Extubation was unsuccessful in 6 out of the 53 children. Reasons for extubation failure in these 6 patients that could have been predicted with a low dTF were: overall muscle weakness in combination with much mucus production, pleural effusion in combination with hyperventilation and exhaustion, bradypnoea with hypercapnia failure. Other reasons for extubation failure, which, however, could not have been predicted with a low dTF were pneumothorax, airway obstruction, and starting non-invasive ventilation after extubation as a precautionary measure to prevent respiratory failure. Details of the extubation failure group are shown in Supplementary Table S4. Three of the 6 children in whom extubation had failed were re-intubated thereafter (respectively within 1 h, after 12 and 27 h after extubation) and three received non-invasive ventilation.

The median dTF values at pre-extubation and post-extubation are presented in Supplementary Tables S3, S4 and Supplementary Figure S3. There was no significant difference between dTF at pre-extubation with PS or with CPAP (*p* = 0.24), and there was no significant difference between dTF after extubation and dTF at pre-extubation (for PS: *p* = 0.25 and for CPAP: *p* = 0.24).

## Secondary outcomes

### Thickening of diaphragm

Tdi-insp and Tdi-exp for baseline, day 4, day 7, day 10, pre-extubation and after extubation are described in Supplementary Table S4. Tdi-insp and Tdi-exp did not change significantly over the duration of ventilation. Overall, at pre-extubation there was no atrophy when considering the baseline thickness (Supplementary Table S4 and Supplementary Figure S5). On the seventh day of ventilation, however, the Tdi-exp of the whole group had decreased 13% versus the baseline value. On day 10, atrophy is no longer apparent (Supplementary Table S5). Seventeen children had developed atrophy at pre-extubation, and extubation failure had occurred in one of them (Supplementary Table S4).

The dosages of sedatives or analgesics in these 17 children were not significantly higher than those of the children without atrophy at pre-extubation.

At baseline and at pre-extubation and post-extubation, there was no significant correlation (after a Bonferroni correction) between age and BSA on the one hand and dTF, Tdi-insp and Tdi-exp on the other hand (Supplementary Table S6).

## Discussion

### Thickening fraction

In this study, we found that the thickening fraction of the diaphragm (dTF) at the time of extubation was not significantly associated with successful extubation in the included ventilated children. From a clinical perspective, this means that the dTF alone cannot predict whether extubation will succeed.

Three of the six children in whom extubation failed had a low dTF (under 10%) in combination with an overall weak state of respiratory muscle function.

We found that most children with a low dTF (10% or lower) before extubation could be successfully extubated. Three children in whom extubation failure occurred had a dTF higher than 10%, which could either reflect greater activity of the diaphragm or high breathing work, which are both possible reasons for extubation failure. Thus so far we demonstrated that a low dTF is not associated with extubation failure, but that the precise cut-off point for successful extubation in either case is not yet clear. Comparing the dTF before extubation for children under 3 months with that of the group above 3 months old revealed no significant difference (*p* = 0.4). Therefore, we assume that with regard to our population the compliant rib cage of infants is not of great influence on dTF when the child is on the ventilator.

Montoro et al. found no correlation between dTF and extubation failure or between dTF and an increased need for non-invasive ventilation post-extubation in ventilated children (20). Twenty of the 47 patients needed NIV after extubation, and extubation failure occurred in 13 of 47 patients. It is not clear whether these 20 patients had received NIV as a precaution or whether they required NIV post-extubation. Montoro et al. found a higher dTF value pre-extubation than in our study; i.e., 30% vs. 15%. Median age, dTF and duration of ventilation (DOV) were the same in both cohorts; the only difference was that Montero and colleagues evaluated dTF during pressure support or spontaneous breathing through an endotracheal tube before extubation. The report makes not clear, however, how many patients received pressure support (and at what level of support) and how many breathed without assist (20).

Our dTF values are more in line with those of Glau et al. with 14.8% pre-extubation in ventilated children. Again, it is not clear how much pressure support was given before extubation (19). IJland et al. found that a median dTF of 15% was associated with successful extubation in 31 ventilated children and found a median dTF of 4% for failure of extubation in three children (21). A recent article of Shah et al. explored the feasibility and utility of using dTF during a spontaneous breathing trial for predicting weaning outcomes in ventilated children with a median age of 11 months (29). Median dTF before extubation with PS 6 above 5 cmH_2_O was 24% (IQR 12–33). One of the 38 children was reintubated and nine others failed a SBT. No information was given about the dTF value of the reintubated patient. The ones who failed the SBT had a significantly lower dTF (12%) versus the successful SBTs (27%). A dTF lower than 25% had a negative predictive value of 94% to predict SBT failure. This is in line with other studies demonstrating a dTF between 21% and 23% (with equivalent PS before extubation) to predict weaning success (25, 26). These rates are higher than in our cohort, maybe because of the effect of giving PS 10 above 5 cm H_2_O before extubation. However, we also found the same values in the 17 children measured with only CPAP (PS 0 above 5 cmH_2_O). dTF could also be down to the level of sedation as Ijland et al. suggested in their study (21). Sedation remains difficult to standardise and compare between studies. More studies are needed to determine the cut-off point of dTF for successful extubation, both for too low and too high values of dTF, with a standardized ventilator support and level of sedation given just before extubation because the type support will influence the dTF. dTF should be measured within the lines of the pleura and peritoneal membrane (30).

### Atrophy

On day 7 of the ventilation period, we found diaphragm atrophy in the whole group, but not on ventilation day 10 and pre-extubation. The explanation for this may be that on day 7 more children were ventilated with a controlled ventilation mode and higher peak pressures and PEEP than on day 10 and pre-extubation. Seventeen out of 53 children (32%) did have atrophy pre-extubation, and extubation failure occurred in one of them. Montoro et al. found diaphragm atrophy in 30 out of 47 patients. A possible explanation for this discrepancy includes their use of NMB in 31 out of 47 patients (20). Glau et al. found atrophy in 19 out of 56 children with NMB (19). We used NMB in only one patient, for 2 days, and found no atrophy in this patient. Glau et al. found an association between diaphragm atrophy and prolonged NIV after extubation (31).

Our results are in line with Vivier et al., who highlighted that diaphragmatic dysfunction assessed by ultrasound in adults does not allow predicting extubation failure. Only ineffective cough was associated with extubation failure in this research (32). This association is also seen in the pediatric study of IJland et al. and in the adult study of Shi et al., in which the expiratory respiratory muscle thickness decreased during mechanical ventilation, and thus coughing power decreased (21, 30).

The question is what dTF really says about diaphragm function and how high or low it should be. Poulard et al. compared transdiaphragmatic pressure with dTF in both healthy and ventilated adults in different breathing conditions, and found a moderately strong correlation in the healthy ones and a weak correlation in the ventilated ones, while large difference within individuals existed (18). Since transdiaphragmatic pressure is usually used as a surrogate for diaphragmatic strength, Poulard and colleagues concluded that ultrasound assessment of diaphragmatic function should be used with caution.

In our cohort we demonstrated an increase in thickness of the diaphragm in 2 of the 6 children who failed extubation, although this was not significantly different from children with extubation success. A recent paper by Shi et al. concluded that an increase in thickness of the diaphragm in ICU patients is usually not due to increase in cross sectional area, but to increase in extracellular matrix (fibrosis or edema) (33). Inspiratory work is redistributed across the various inspiratory muscles and this redistribution could have played a role in the high inter-individual variability of dTF in this study and transdiaphragmatic pressure or esophageal pressure does not solely depend on diaphragm activation. Extra-diaphragmatic muscles such as the parasternal intercostal muscles also thicken during inspiration in healthy and ventilated patients (34, 35). Weakness of the diaphragm may be compensated with parasternal or expiratory muscle activity (21, 36, 37). IJland et al. measured thickness of expiratory muscles in children and found a rapidly developing change in diaphragm and expiratory muscles thickness after initiation of mechanical ventilation, but changes in thickness of the diaphragm and expiratory muscles were not significantly correlated. The decrease in expiratory muscles thickness was significantly higher in the three patients in whom extubation failure occurred than in the successful extubation cases in this study (21).

In the whole group, twenty-three children showed limb muscle weakness after their illness, but only two of them had failed extubation. These two children had appeared weak and were treated by the pediatric physiotherapist. Apparently, limb muscle weakness does not equal diaphragmatic atrophy. ICU-acquired muscle weakness (ICU-AW) is not often diagnosed in children unlike in adults (38–40). A retrospective cohort study with 200,000 PICU admissions found an incidence ICU-AW of 0.02%, and ICU-AW was more frequently reported in the children with admission diagnoses of respiratory illness and infection. Furthermore, ICU-AW was associated with a longer length of stay, longer duration of ventilation and longer duration of chronic care (40). Dres et al. found only limited overlap between ICU-AW and diaphragm dysfunction in adult critically ill patients (15). Diaphragm dysfunction in that study was twice as frequent as ICU-AW and had a direct negative impact on weaning outcome.

For future research, assessing all respiratory muscles in children who are prone to muscle atrophy is advisable or should be considered, as Tuinman et al. suggested (37). By assessing all respiratory muscles with ultrasound, we might have had a good explanation for and at least a completer view of the failed extubations in the five children without upper airway problems in our study. However, although a recent study shows that ultrasound assessment of the parasternal intercostal muscle is feasible in the adult intensive care unit (41) and one study assessed abdominal muscles in ventilated children (21), more studies of ultrasounds of the intercostal and abdominal muscles are needed in ventilated and healthy children before this approach can be used in clinical practice.

The strength of this study is that we looked specifically at the predictive value of dTF on extubation success and failure (not many pediatric studies have done that) and that we also looked at post-extubation, which not many studies have done either. We used a standardized ventilation protocol and also measured 17 patients with CPAP before extubation, which has never been done before.

Some limitations can be identified. First, this was a small and heterogeneous patient population, and extubation failure occurred in only six patients (11.3%). Multivariate analyses were not feasible due to low sample size. We would have done better to base the sample size on a sample calculation instead of taking a convenience sample. Second, due to the occasional absence of the two observers some ultrasounds were missed, this could have influenced the results. Third, the method of diaphragm measurement may not have been the optimal one. The current consensus is to measure just the muscle within the lines of the fascia. This knowledge was not yet available at the beginning of our study (42, 43). The reason is that because membranes are biologically active, mechanical forces related to ventilation can lead to inflammation with remodeling and thickening of the tissues. This remodeling of the tissues may have caused variation in size of thickness of the diaphragm (37, 42, 44). Fourth, we measured dTf in 17 children on CPAP before extubation and saw no significant difference in dTF for CPAP versus PS. As Khemani and colleagues have shown that CPAP before extubation better predicts whether extubation could be succesful, maybe we would have done better to set all children on CPAP before extubation (45). Nevertheless, following our study protocol we standardly applied PS 10 above 5 cmH_2_O. We only set CPAP when there was time for extubation and the child tolerated it. For future research, it would be better to commence the children on CPAP before extubation as Khemani et al. suggested and then measure dTF (45). Fifth, with the use of a high frequency (4–10 MHz) linear array transducer the resolution and margin of effort of our measurements are to be taken into account.

For future studies on ultrasounds of the diaphragm, we recommend to measure the diaphragm and extra-diaphragmatic muscles of larger groups of ventilated children and using CPAP before extubation, which could allow to validly compare the successful extubations with the extubation failures. The focus should be on patients with muscle diseases, or with a chronic condition, abnormal lung function, poor nutritional status, or on disabled children who are on the ventilator for 5 days or more. The extubation failure group should be divided into groups of different causes of extubation failure, in order to find out whether after extubation with ultrasound a good outcome can be predicted. In this research the consensus method of ultrasound should be used, as well as a standardized method with standard mechanical ventilation settings and sedation levels (42).

## Conclusion

There was no significant association between thickening fraction of the diaphragm and extubation success in ventilated children.

## Data Availability

The original contributions presented in the study are included in the article/**Supplementary Material**, further inquiries can be directed to the corresponding author.
