# Supplementary material for: No association between thickening fraction of the diaphragm and extubation success in ventilated children
Source: Front Pediatr. 2023 Mar 24;11:1147309. doi: 10.3389/fped.2023.1147309 (PMC10081691; doi:10.3389/fped.2023.1147309)
Supplement: Supplementary file 1 [file Datasheet1.pdf]

**Supplemental file 2. Protocol for determining type of weaning**

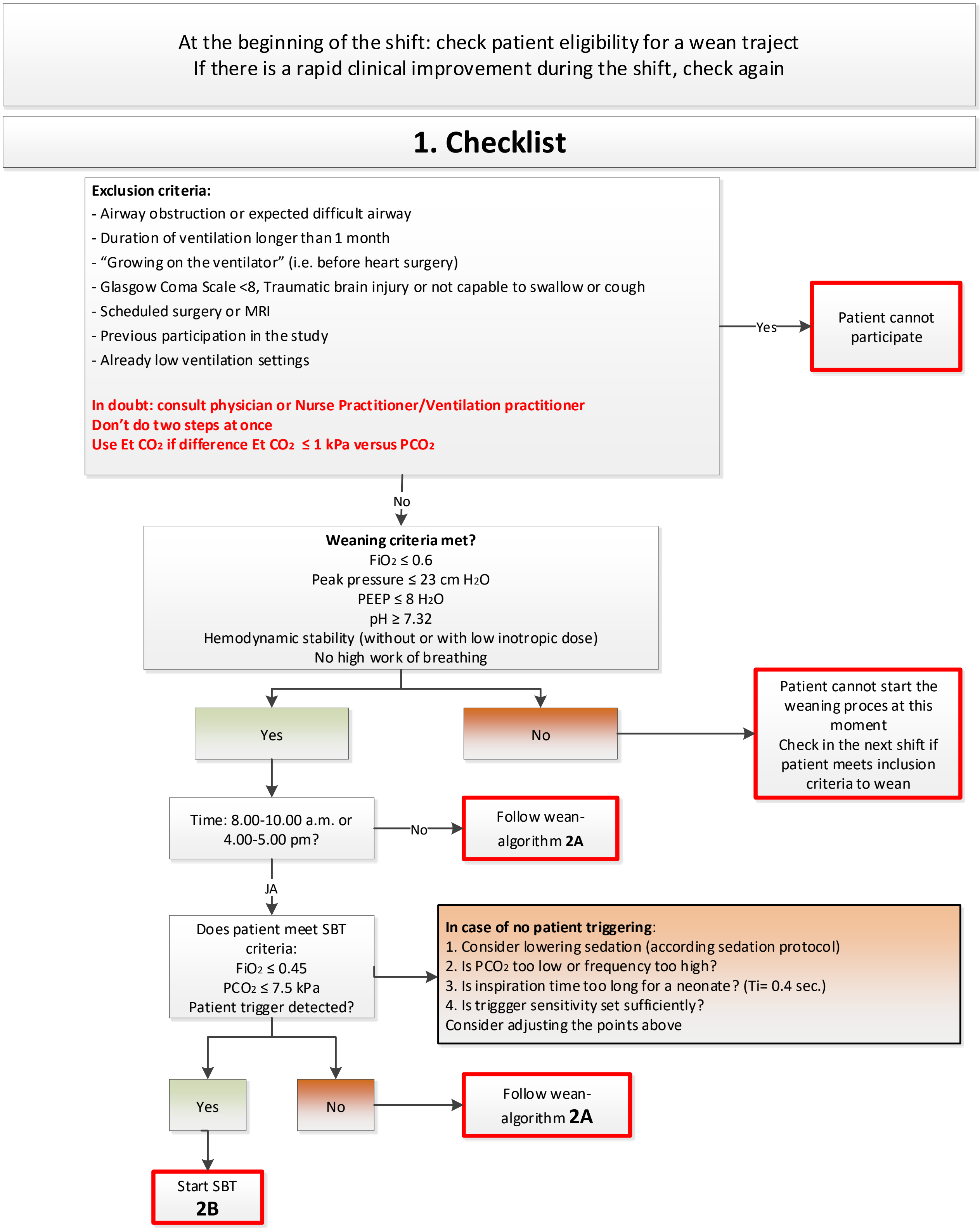

## Supplemental file 3. Weaning algorithm

# 2A. Weaning Algorithm

$FiO_2$ based on saturation: lower oxygen in steps of 5%, a 5 minutes till $F_iO_2$ = 0.4

Lower PEEP level every hour with 1 cm $H_2O$ based on saturation till PEEP ≤ 5 cm $H_2O$
In case of more oxygen requirement after lowering PEEP: consult physician or practitioner

PC and triggering:
Switch to Pressure Support*

Based on $EtCO_2$ or $PCO_2$ (< 7.5 kPa):
Lower PS above PEEP with steps of 2 cm $H_2O$ per hour until tidal volume is 4 ml/kg (ideal weight)
Or till PS has a minimum of 8 cm $H_2O$ above PEEP

PRVC and triggering:
Switch to Volume Support*

Based on $EtCO_2$ of $PCO_2$ (< 7.5 kPa):
Lower TV with steps of 1 ml/kg until 4 ml/kg (ideal weight)

**In case of no patient triggering**:
1. Consider lowering sedation (according sedation protocol)
2. Is $PCO_2$ too low or frequency too high?
3. Is Inspiration time too long for a neonate? (Ti= 0.4 sec.)
4. Is trigger sensitivity set sufficiently?

Consider changing the points above

Target reached?

**Extubation criteria**
No high work of breathing
$FiO_2$ ≤ 0.4
pH > 7.32/ $PCO_2$ <7.5
$SpO_2$ > 95% or > 75% in case of cyanotic heart disease or > 88% in case of chronic lung disease or > 92% in case of ARDS.
EMV > 8, capable to swallow or cough
Mucus production ≤ suction 3 x per hour
Hemodynamically stable (with no or low inotropic dose)
No new fever episode> 38.5 degrees
No scheduled surgery/MRI < 24 hrs
No clinical signs of edema

Yes

Stop feeding
(2 hours at duodenal feeding and 4 hours at stomach feeding)
Dexamethasone needed?

Inform the physician and discuss the time of extubation

**EXTUBATION**

No

In case of failing weaning, go back to basic values
Use the check list again in the next shift

* At Pressure Support or Volume Support:
Set inspiration cycle off (ICO) at 5-10% in neonates (higher in case of leakage) and at 30% in children > 6 months
Set Trigger time out (TTO) at 3-4 sec. in neonates until a year old and 5-7 sec. in children

*AMC:
P-CMV+ or P-SIMV+ or if possible, Spontaneous mode
In case of tachypnea in Spontaneous mode and children < 1 year old : go back to P-CMV+ mode and lower frequency until patient is triggering well (don't lower less than a frequency of 15 p/m).
Set an apnea time of 5 seconds

## Supplemental file 4. Spontaneous Breathing Trial (SBT)

### 2B. Spontaneous Breathing Trial (SBT)

**Start weaning in day shift between 8.00 en 10.00 a.m. and at 4.00 p.m.**

**Start SBT**
Set ventilation mode to Pressure Support
PS 10 $cmH_2O$
PEEP 5 $cmH_2O$;
$FiO_2$ keep at the same level

↓

Testing for 2 hours
Assess per 15 minutes: Is the child able to breathe adequately by himself
*If necessary, agree on a longer time frame with the physician for better assessment*

Observe: heart rate/work of breathing/comfort/ blood pressure.
Take a blood gas sample after 2 hours and assess Et $CO_2$
(Use Et $CO_2$ only if Et $CO_2$ corresponds to $PCO_2$ with maximum difference of 1 kPa)

↓

**SBT is a success and extubation criteria have been met in case of:**

| | | |
|---|---|---|
| No high work of breathing | $FiO_2 \leq 0.4$ | pH > 7.32/ $PCO_2$ <7.5 |
| $SpO_2$ > 95%, > 75%, cardiac > 88%, CLD >92%, ARDS | HF < 20% increase compared to basic value | BP ≤ 10% increase compared to basic value |
| EMV > 8, inable to swallow or cough | Mucus suction ≤ 3 x per hour | Hemodynamically stable |
| No new fever episode> 38.5 degrees | No scheduled surgery/MRI < 24 u | No clinical signs of edema |

**SBT succeeded?**
Continue PS 10/5 and prepare on extubation.

↓

Stop feeding (2 hours at duodenal feeding and 4 hours at stomach feeding) Dexamethasone needed?

↓

Inform the physician and discuss time of extubation → **EXTUBATION**

**SBT failed?**
Go back to settings before start SBT and start weaning algorithm (2A) or check patient again with the checklist (1)

*** At Pressure Support:**
Set inspiration cycle off (ICO) at 5-10% in neonates (higher in case of leakage) and at 30% in children > 6 months
Set Trigger time out (TTO) at 3-4 sec. in neonates until a year old and 5-7 sec. in older children

*Explanation Nurse-led weaning protocol, supplemental file 2-4*

*1. Checklist start criteria:* Weaning can be started at any time of the day if the patient meets the start criteria according the checklist (supplemental file 1). PEEP is being decreased in steps of 1 $cmH_2O$ every hour when $FiO_2$ is ≤ 40%. If more oxygen is required when PEEP is being decreased, the nurse should consult a physician, nurse practitioner or ventilation practitioner (registered nurse with additional training in ventilation management) about the necessity of increasing PEEP. The saturation limit is ≥ 95% for most children, but ≥ 88% for children with chronic lung disease, ≥ 92% for children with ARDS and ≥ 75% for cardiac patients with mixed circulation. (18)

*2. Wean algorithm:* If the patient breathes spontaneously ('triggers the ventilator'), the wean algorithm can be followed (supplemental file 2). The ventilation mode is set to a support mode; for the Servo i, the 'auto-mode' mode (with pressure support (PS) for pressure control (PC) and volume support (VS) for pressure regulated volume control (PRVC); for the Hamilton the PS mode. If the patient does not trigger the ventilator, the possible reason and a solution have to be found to stimulate triggering. In the Amsterdam UMC, the mode was set to PC or PC-SIMV if the support mode led to tachypnea, and the frequency was lowered until the patient was triggering well. PS above PEEP can be reduced to a minimum of 8 $cmH_2O$ in one-hour steps of 2 $cmH_2O$ above PEEP, or the tidal volume can be reduced to a minimum of 4 ml/kg.

*3: Spontaneous Breathing Trial* (Supplemental file 3). For patients meeting the start criteria, an SBT was planned between 8 and 10 a.m. and again in the afternoon around 4 and 6 p.m., with the following ventilation settings during 2 hours: PS 10 $cmH_2O$ above PEEP 5 $cmH_2O$, and $FiO_2$ equal to the start of the SBT. During the SBT, the patient was observed to see whether he or she could breathe calmly with the predefined range for respiratory rate, with stop criteria if the SBT fails.
