# Supplementary figures and images for: No association between thickening fraction of the diaphragm and extubation success in ventilated children
Source: Front Pediatr. 2023 Mar 24;11:1147309. doi: 10.3389/fped.2023.1147309 (PMC10081691; doi:10.3389/fped.2023.1147309)

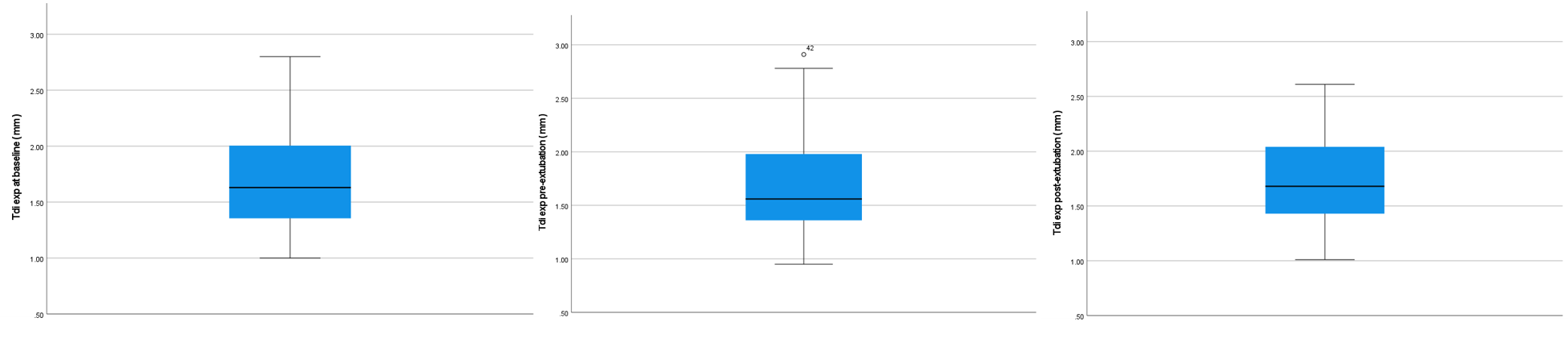

Supplement: Supplementary file 2 [file Image1.png]

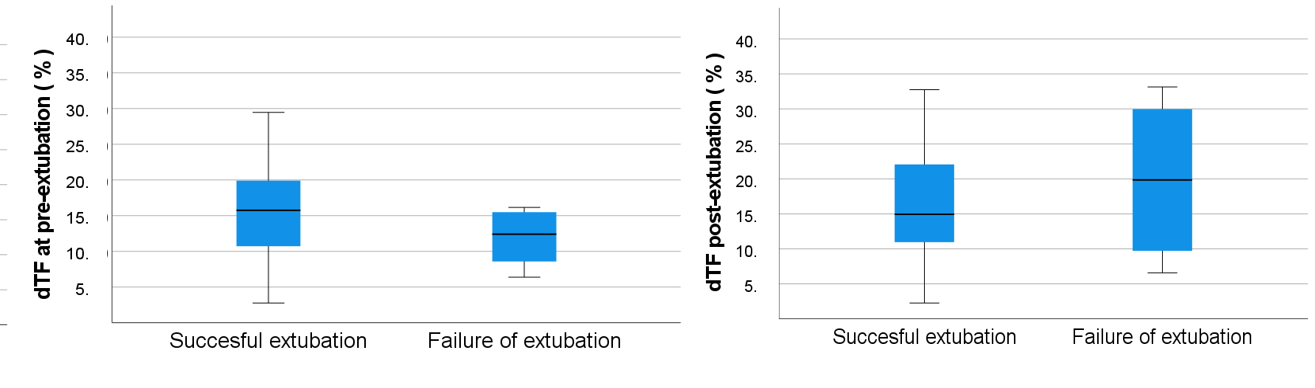

Supplement: Supplementary file 3 [file Image2.png]
