# Supplementary material for: No association between thickening fraction of the diaphragm and extubation success in ventilated children
Source: Front Pediatr. 2023 Mar 24;11:1147309. doi: 10.3389/fped.2023.1147309 (PMC10081691; doi:10.3389/fped.2023.1147309)
Supplement: Supplementary file 4 [file Table1.docx]

**Table 1. Ultrasound schedule**

| Duration of ventilation | Day 1 | Day 4 | Day 7 | Day 10 | On the day of extubation  After start SBT or already on a support mode | Just before extubation on CPAP | Within 24 hours of  extubation |
| --- | --- | --- | --- | --- | --- | --- | --- |
| > 48 hours <  4 days | X |  |  |  | X | X | X |
| 5-6 days | X | X |  |  | X | X | X |
| > 6 days <10 days | X | X | X |  | X | X | X |
| >10 days | X | X | X | X | X | X | X |
