# Supplementary material for: No association between thickening fraction of the diaphragm and extubation success in ventilated children
Source: Front Pediatr. 2023 Mar 24;11:1147309. doi: 10.3389/fped.2023.1147309 (PMC10081691; doi:10.3389/fped.2023.1147309)
Supplement: Supplementary file 5 [file Table2.docx]

**Additional file 8. Table 4. Cases of failed extubation**

| **case** | **1. (reintubation)** | **2.(reintubation)** | **3. (reintubation)** | **4.(NIV)** | **5. (NIV)** | **6. (NIV)** |
| --- | --- | --- | --- | --- | --- | --- |
| **Age (months)** | 1 | 180 | 0.2 | 192 | 3 | 1 |
| **Reason of admission** | Respiratory insufficiency (bronchiolitis) | Trauma | Post-operative  (correction oesophageal atresia) | Deterioration in Myasthenia Gravis | Pneumonia and pleural effusion | Coronary clot and heart failure |
| **DOV (days)** | 180.0* | 15.1 | 24.6 | 36.0 | 8.9 | 48.0 |
| **Cause of failure** | Airway obstruction, mucus and stridor | Pleural effusion  Anxiety, hyperventilation and exhaustion | Pneumothorax | Overall weakness and much mucus | Bradypnoea and weakness based on high doses of pyridoxal phosphate because of a PNPO deficiency | NIV was a precautionary measure to prevent respiratory failure |
| **dTF at CPAP before extubation** | NA | 6.5% | 15.5% | NA | NA | NA |
| **dTF at pressure support before extubation** | 16.2% | 9.8% | 14.3% | 8.6% | 6.4% | 15.1% |
| **atrophy** | no | -26.8% | no | no | no | no |
| **Increase of Tdi-exp before extubation** | +38.0% |  | no | no | no | +21.0% |
| **Sedation before extubation** | Midazolam  100 mcg/kg/hour | Midazolam  8 mcg/kg/hour  Remifentanil  0.22 mcg/kg/hour | no | Remifentanil  0.15 mcg/kg/hour | Midazolam  42 mcg/kg/hour | no |

NIV: non-invasive ventilation; NA: not available; * this patient needed a trachea cannula and prolonged ventilation
