# Supplementary material for: No association between thickening fraction of the diaphragm and extubation success in ventilated children
Source: Front Pediatr. 2023 Mar 24;11:1147309. doi: 10.3389/fped.2023.1147309 (PMC10081691; doi:10.3389/fped.2023.1147309)
Supplement: Supplementary file 6 [file Table3.docx]

**Additional file 9. Table 5. Measurements of dTF, Tdi insp, Tdi exp over time**

|  | **Baseline**  **n=52** | **Day 4**  **n=28** | **Day 7**  **n=14** | **Day 10**  **n=6** | **Pre-extubation**  **PS**  **n=50** | **Pre-extubation**  **CPAP**  **n=17** | **Post- extubation**  **n=51** | p*  *pre-extubation (PS 10/5 cmH_2_O) vs baseline* | p*  *pre-extubation (CPAP 5 cmH_2_O) vs baseline* | *p**  *post-extubation vs baseline* |
| --- | --- | --- | --- | --- | --- | --- | --- | --- | --- | --- |
| **dTF***  **Thickening fraction (%)** |  |  |  |  | 15.2  (9.7-19.3) | 16.1  (12.3-23.2) | 14.9  (10.9-23.4) |  |  |  |
| **Tdi end-inspiration***  **(mm)** | 1.82  (1.49-1.82) | 1.70  (1.52-1.94) | 1.60  (1.44-2.10) | 1.58  (1.17-2.20) | 1.83  (1.60-2.30) | 1.78  (1.50-2.59) | 1.95  (1.71-2.38) | *0.46* | *0.25* | *0.66* |
| **Tdi end-expiration***  **(mm)** | 1.63  (1.35-2.02) | 1.51  (1.41-1.72) | 1.44  (1.28-1.96) | 1.51  (1.05-2.10) | 1.56  (1.36-2.00) | 1.53  (1.22-2.13) | 1.68  (1.43-2.08) | *0.24* | *0.25* | *0.18* |
| **Atrophy (%) **** |  | -7.9  (-30.0 vs 6.0) | -13.2  (-7.0 vs -6.0) | -7.9  (-30.0 vs 8.0) | -4.5  (-2.0 vs 1.0) | -6.5  (-13.0 vs 11.0) |  |  |  |  |

*Median (IQR); p*= Paired samples t test; ** Difference Tdi end-expiration versus Tdi end-expiration at baseline
