# Supplementary material for: No association between thickening fraction of the diaphragm and extubation success in ventilated children
Source: Front Pediatr. 2023 Mar 24;11:1147309. doi: 10.3389/fped.2023.1147309 (PMC10081691; doi:10.3389/fped.2023.1147309)
Supplement: Supplementary file 7 [file Table4.docx]

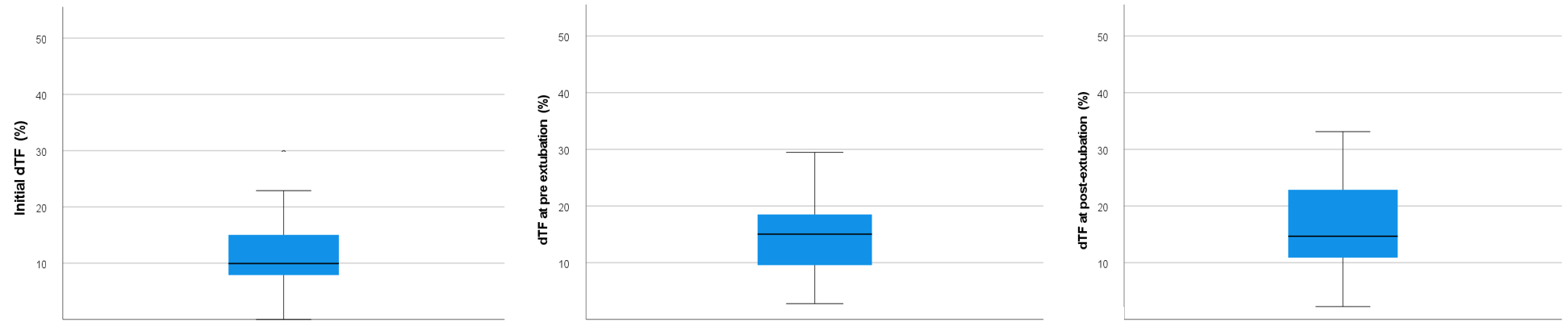


dTF= thickening fraction of the diaphragm; Initial dTF, n=52 ; dTF at pre-extubation, n=50 ; dTF at post-extubation, n=51

**Additional file 10. Figure 4. Thickening fraction at pre-extubation and at post-extubation**
