# Supplementary material for: No association between thickening fraction of the diaphragm and extubation success in ventilated children
Source: Front Pediatr. 2023 Mar 24;11:1147309. doi: 10.3389/fped.2023.1147309 (PMC10081691; doi:10.3389/fped.2023.1147309)
Supplement: Supplementary file 8 [file Table5.docx]

**Additional file 12. Table 6. Spearman correlations between age, body surface area and Tdi and dTF in time**

|  | BSA (m2 | Age in months |
| --- | --- | --- |
| dTF Pre-extubation | r= -0.242 n=52 p=0.10  95% CI -0.501 / 0.058 | r= -0.219 n=50 p=0.14  95% CI -0.373 / 0.201 |
| dTF Post-extubation | r= -0.256 n=52 p=0.08  95% CI -0.507 / 0.036 | r= -0.320 n=51 p=0.02  95% CI -0.555 / -0.037 |
| Tdi insp baseline | r= 0.241 n=52 p=0.09  95% CI -0.046 / 0.491 | r= 0.113 n=52 p=0.42  95% CI -0.173 / 0.382 |
| Tdi insp Pre-extubation | r= -0.087 n=52 p=0.56  95% CI -0.366 / 0.208 | r= -0.187 n=50 p=0.19  95% CI -0.449 / 0.105 |
| Tdi insp Post-extubation | r= -0.217 n=52 p=0.13  95% CI -0.474 / 0.073 | r= -0.323 n=51 p=0.02  95% CI -0.556 / -0.044 |
| Tdi exp baseline | r= 0.256 n=51 p=0.07  95% CI -0.03 / 0.503 | r= 0.143 n=52 p=0.31  95% CI -0.143 / 0.408 |
| Tdi exp Pre-extubation | r= 0.020 n=48 p=0.89  95% CI -0.273 / 0.310 | r= -0.094 n=49 p=0.52  95% CI -0.373 / 0.201 |
| Tdi exp Post-extubation | r= -0.100 n=50 p=0.50  95% CI -0.375 / 0.192 | r= -0.201 n=51 p=0.16  95% CI -0.458 / 0.087 |

r= correlation coefficient; dTF= thickening fraction of the diaphragm; Tdi insp.= thickening fraction at end-inspiration; Tdi exp. = thickening fraction at end-expiration, BSA = bodysurface area.

Bonferoni corrected p-value = 0.0063
