# Supplementary material for: No association between thickening fraction of the diaphragm and extubation success in ventilated children
Source: Front Pediatr. 2023 Mar 24;11:1147309. doi: 10.3389/fped.2023.1147309 (PMC10081691; doi:10.3389/fped.2023.1147309)
Supplement: Supplementary file 10 [file Table7.docx]

**Table 2. Patient characteristics**

| **Characteristics (n=53)** | **Total: n=53** | **Successful extubation**  **n=47** | **Failed extubation**  **n=6** | **P value*** |
| --- | --- | --- | --- | --- |
| Age (months)* | 3.0 (0.1-66.0) | 3.0 (0.10-48.0) | 2.0 (0.81-183.0) | 0.045 |
| Weight (kg)*  Height (cm)*  Body surface*(m^2^) | 4.95 (3.3-24.5)  56.0 (50.5-130.5)  0.28 (0.2-0.9) | 5.20 (3.0-23.5)  56.0 (50.0-130.0)  0.28 (0.20-0.9) | 4.73 (4.2-62.0)  59.0 (54.3-162.5)  0.27 (0.2-1.7) | 0.49 |
| Sex, n (%)  Female | 22 (41.5%) | 19 (40.0%) | 3 (50.0%) | 0.49 |
| PRISM III* | 14.0 (8.0-16.5) | 14.0 (8.0-16.0) | 13.5 (9.0-19.8) | 0.42 |
| **Reason for ventilation**   - Respiratory insufficiency - Postoperative - Pulmonary hypertension - Traumatic brain injury and trauma - Neuromuscular disease - Sepsis - Other | 14 (26.4%)  19 (35.8%)  3 (5.7%)  7 (13.2%  3 (5.7%)  1 (1.9%)  6 (11.3%) | 12 (25.5%)  18 (38.3%)  3 (6.4%)  6 (12.8%)  2 (4.3%)  1 (2.1%)  5 (10.6%) | 2 (33.3%)  1 (16.7%)  0  1 (16.7%)  1 (16.7%)  0  1 (16.7%) | 0.80 |
| **Initial ventilation settings***  PIP (cmH2O)  PEEP(cmH2O)  FiO_2_ (%) | 12.0 (10.0-14.0)  6.0 (5.0-8.0)  0.33 (0.3-0.5) | 12.0 (9.8-14.0)  5.0 (5.0-8.0)  0.33 (0.3-0.5) | 13.0 (9.5-15.0)  7.5 (5.0-9.3)  0.3 (0.3-0.6) | 0.28  0.19  0.20 |
| Duration of ventilation (hours)* | 114.0 (55.5 - 193.5) | 114.0 (56.5-190.3) | 145.5 (48.0-327.3) | 0.73 |
| Length of Stay ICU? (days)* | 11.5 (6.7-19.9) | 10.2 (6.6-19.5) | 30.3 (13.6-81.0) | 0.32 |
| **Doses of sedatives and opioids at time of extubation***  Midazolam mcg/kg/hrs  Morphine mcg/kg/hrs  Ketamine mg/kg/hrs  Clonidine mcg/kg/hrs  Remifentanyl mcg/kg/hrs  Fentanyl mcg/kg/hrs | 71.5 (43.0 – 143.3) n=28  5.8 (5.0-10.0) n=16  0.5 (0.3-2.1) n=6  0.7 (0.2 – 1.8) n=13  0.15 (0.1-0.2) n=3  2.5 n=1 | 78.0 (48.0-175.0) n=25  5.8 (5.0-10.0) n=16  0.5 (0.3-2.1) n=6  0.7 (0.2-1.8) n=7  0.1 n=1  2.5 n=1 | 42.0 (8.0-42.0) n=3  0.2 (0.2) n=2 |  |
| Dexamethasone before extubation (n) | 7 | 6 | 1 |  |
| Reintubation | 3 (5.7%) |  |  |  |
| High flow nasal cannula (therapeutic) | 17 (32.1%) |  |  |  |

*Median (IQR); p* successful extubation versus failed extubation; FiO_2_; fraction of inspired oxygen, PIP; peak inspiratory pressure, PEEP; positive end expiratory pressure, PRISM; pediatric risk of mortality
